# Regeneration of Bone, Cartilage, and Tooth Following Lower Jaw Amputation in Newts

**DOI:** 10.3390/biomedicines14020434

**Published:** 2026-02-14

**Authors:** Kento Tsubosaki, Taisuke Hani, Kazuya Fujita, Kaori Sato, Tomoo Kudo, Yuuichi Soeno, Tatsuyuki Ishii, Kazuo Kishi, Chikafumi Chiba, Yuji Taya

**Affiliations:** 1 Department of Pathology, The Nippon Dental University School of Life Dentistry at Tokyo, 1-9-20 Fujimi, Chiyoda-ku, Tokyo 102-8159, Japan; tsubosaki@tky.ndu.ac.jp (K.T.); t-hani@tky.ndu.ac.jp (T.H.); kazuyafujita@hotmail.com (K.F.); k-sato@tky.ndu.ac.jp (K.S.); tkudo@tky.ndu.ac.jp (T.K.); soeno-path@tky.ndu.ac.jp (Y.S.); 2 Department of Plastic and Reconstructive Surgery, Keio University School of Medicine, 35 Shinanomachi, Shinjuku-ku, Tokyo 160-8582, Japan; ttsyksh@gmail.com (T.I.); kkishi@keio.jp (K.K.); 3 Institute of Life and Environmental Sciences, University of Tsukuba, Tennodai, Tsukuba 305-8572, Japan; 4 First-Year Experience, The Nippon Dental University School of Life Dentistry at Tokyo, 1-9-20 Fujimi, Chiyoda-ku, Tokyo 102-8159, Japan

**Keywords:** newt, regeneration, lower jaw, bone, Meckel’s cartilage, tooth

## Abstract

**Background/Objectives**: In humans, diseases such as oral cancer may require surgical amputation of the jaw. This severe disruption causes impairments in eating, swallowing, and speech, leading to a significant decline in quality of life. In contrast, newts, a group of urodele amphibians, can regenerate their jaws even in adulthood. This study explored how adult newts reconstruct lower jaws after substantial loss and clarified how this process contributes to rapid functional recovery when feeding becomes impossible. **Methods**: Adult Japanese fire-bellied newts (*Cynops pyrrhogaster*) underwent surgical amputation of the anterior half of their lower jaws. Regeneration was monitored for 64 weeks using histological analyses of bone, cartilage, and dental tissues and micro-computed tomography (micro-CT)-based osteomorphometry to quantify structural changes in the regenerating lower jaw. **Results**: Histological observations and osteomorphometry revealed the following: epithelial coverage of the amputation margin; ectopic cartilage formation, growth, and regression; bone resorption at the amputation margin prior to bone regeneration; anterior extension of the lower jaw bone along the original dentition position, followed by its thickening; and dental lamina invagination with tooth germ formation. Through these processes, the lower jaw bone, Meckel’s cartilage, and dentition were restored by 64 weeks post-amputation to their pre-amputation states. **Conclusions**: This study delineates the full sequence of lower jaw regeneration in adult newts, demonstrating complete restoration of bone, cartilage, and teeth after substantial lower jaw loss. These findings provide a detailed framework for understanding urodele jaw regeneration and may inform future strategies for promoting jaw reconstruction in humans.

## 1. Introduction

In mammals, including humans, the jaw does not regenerate when lost [1,2]. Therefore, reconstructive surgery is required in cases of severe jaw trauma or oral cancer resulting in adult human jaw loss. This surgery can include transplanting scapula or fibula tissue to the defect site or reinforcing the jaw with metal plates. These procedures usually lead to jaw deformity and impaired mastication and speech, thereby reducing the quality of life [3].

In contrast, newts, a group of urodeles belonging to the family Salamandridae, possess exceptionally high regenerative capabilities among tetrapods. Even terrestrial adults adapted to land environments can regenerate various body parts, including parts of limbs, tails, and jaws [4,5,6,7,8,9]. Among amphibians, anurans possess the ability to regenerate during the larval stage but lose it in adulthood [7,10]. Axolotls (*Ambystoma mexicanum*) exhibit neoteny, and their regenerative capacity diminishes as they develop into adulthood. Therefore, a newt’s regenerative capacity does not diminish with maturity, making it an excellent experimental animal model for tissue regeneration. If human jaw regeneration is possible, as in newts, it could help maintain quality of life.

Regarding the regeneration of newt jaws, Kurosaka et al. [11] observed specimens up to 180 days after half amputation of the anterior lower jaw. Under stereomicroscopy, regeneration appeared largely complete, with the lower jaw bones fused at the midline and tooth regeneration observed in a narrow area. However, knowledge regarding the regenerative processes of newt jaws remains limited, with many unresolved aspects. Therefore, this study focused on the regeneration processes following the amputation of the anterior half of the lower jaw of the Japanese fire-bellied newt (*Cynops pyrrhogaster*), specifically examining the regeneration of the bone and cartilage forming the jaw skeleton and the teeth forming dentition. The results showed that by 64 weeks post-amputation, the lower jaw bone, Meckel’s cartilage, and dentition had restored to their pre-amputation state. This study discusses how the regeneration process of the lower jaw makes sense as a strategy for newts to adapt quickly when they are unable to feed due to the loss of their lower jaw.

## 2. Materials and Methods

### 2.1. Animals

Adult male Japanese fire-bellied newts *Cynops pyrrhogaster*, a species endemic to Japan, were provided by Prof. Chikafumi Chiba (Institute of Life and Environmental Sciences, University of Tsukuba). Identification of individual newts was based on differences in the red and black patterns on the ventral surface. Newts were kept at room temperature (approximately 20 °C) under a 12-h light/dark cycle and fed five times a week. Newts were kept individually in plastic containers, with a resting place (land) provided in shallow water. Healthy animals were randomly selected from among individuals that were not underweight for use in the experiments. Animals with almost the same proportion and size (total body length: 12–13 cm; estimated age: >5 years) were selectively used. A total of 77 newts were used in the experiment (74 for micro-computed tomography (micro-CT) analysis, 25 for osteomorphometry, and 27 for histological observation; some individuals overlapped between these categories) (Supplementary Table S1). Based on preliminary calculations of sample size adequacy, we estimated that approximately 80 animals would be required and prepared that number for the study. At each time point examined, three or more animals were used in both the control and experimental groups. For the endpoint, the abdomen was injected with a higher dose of FA100. This animal experiment was conducted with the approval of the Animal Practice Committee of the Nippon Dental University School of Life and Dentistry at Tokyo (approval numbers 19-04-1, 22-14 and 24-17).

### 2.2. Surgical Lower Jaw Amputation

**Figure 1:**
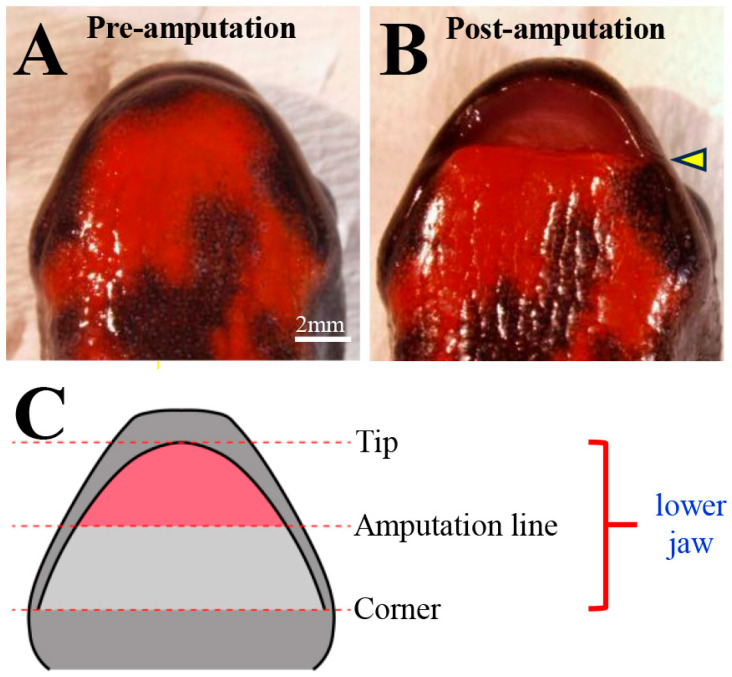
Lower jaw amputation. Stereoscopic images (ventral view) of pre-amputation (**A**) and post-amputation (**B**) newts. Schematic diagram of the standardized amputation line and amputation area (red area) (**C**). The amputation site is indicated by an arrowhead. Scale bar: 2 mm.

Newts were immersed in a 0.1% solution of FA100 (DS Pharma Animal Health Co., Ltd., Osaka, Japan) for 1 h to achieve general anesthesia. After rinsing, the anterior half of the lower jaw was amputated using surgical scissors under a stereomicroscope. Figure 1 shows stereomicroscope images (ventral view) before and after lower jaw amputation, as well as a schematic diagram of the standardized amputation area. Anatomically, the amputation line corresponded to a position slightly more anterior than the tip of prearticular bone. Therefore, the amputation was limited to the dentary bone.

At the end of the experimental period, the animals were euthanized via decapitation under general anesthesia. The heads, including the lower jaw, were fixed in 4% paraformaldehyde phosphate buffer (Fujifilm Wako Pure Chemicals Corp., Osaka, Japan) at 4 °C for 20 h. The lower jaw before amputation and the states of lower jaw regeneration after amputation and immediately before euthanasia were recorded with a digital camera attached to a stereomicroscope (DS-Fi3, SMZ745T; Nikon Corp., Tokyo, Japan).

### 2.3. Three-Dimensional Structural Analysis Using Micro-Computed Tomography and Measurement of Bone Morphology

Before and after lower jaw amputation and at a defined period of the experiment, newts were imaged under general anesthesia via micro-CT (ScanXmate-D100SS270; Comscan Techno Corp., Kanagawa, Japan). During imaging, welfare measures were taken to reduce harm to the newts, such as preventing them from drying out. The obtained data were reconstructed with coneCTexpress software (2.3.1.0, Voxel Works Co., Ltd., Tokyo, Japan), and three-dimensional construction and morphometry of bone tissues were performed using TRI/3D-BON-FCS64 software (12.1.0.0, BON-FCS, RATOC Systems, Inc., Tokyo, Japan).

Osteomorphometric bone parameters, including regenerated and existing bone and regenerated bone parameters alone, morphometric measurements of bone volume (BV), bone surface area (BS), and the bone surface-to-volume ratio (BS/BV), were quantified. Values for the regenerated bone alone were calculated by subtracting the parameter values for the bone immediately after amputation from total bone parameter values. The total bone volume (TBV), total bone surface area (TBS), and total bone surface-to-volume ratio (TBS/TBV) were measured for both the existing lower jaw bone and the newly regenerated bone combined. The regenerative bone volume (RBV), regenerative bone surface area (RBS), and regenerative bone surface-to-volume ratio (RBS/RBV) were calculated for regenerated bone alone. The lower jaw bone of the newt, including regenerated bone, did not exhibit the cylindrical structure typical of long bones commonly used in osteometry. Therefore, extraction and measurement were performed without distinguishing between compact bone and cancellous bone. CT grayscale values were converted to tissue mineral density (TMD, mgHA/cm^3^) using a hydroxyapatite phantom manufactured by RATOC Systems, Inc. A TMD threshold of 200 mgHA/cm^3^, corresponding to the inclusion of early regenerated bone, was consistently applied to all specimens. Furthermore, measurements were performed after removing residual non-lower jaw elements.

Because the present study was designed as an exploratory investigation focusing on the most informative findings identified thus far, the statistical strength of the analyses may be constrained. Nonetheless, all analyses were conducted using the samples available at each time point. Quantitative measurements obtained from independent animal groups were analyzed using one-way analysis of variance, followed by pairwise comparisons using the Tukey–Kramer method, with a significance level of 0.05. All statistical analyses were performed using SPSS software (version 28.0.1; IBM Corp., New York, NY, USA).

### 2.4. Tissue Preparation for Histological Analysis

The lower jaw, separated from the newt’s head, was demineralized at 4 °C in a 10% ethylenediaminetetraacetic acid (EDTA 2Na, 345-01865, FUJIFILM Wako Pure Chemical Corporation, Osaka, Japan) solution at pH 7.0. Then, it was split along the midline. After embedding, sections were cut using a rotary microtome (HM355; PHC Holdings Co., Ltd., Tokyo, Japan) equipped with a section transfer system. Masson–Goldner (MG) staining was performed using the Masson–Goldner staining kit (1.00485.0001, Merck, Darmstadt, Germany) according to the manufacturer’s instructions. Alcian blue (AB) staining was performed by incubating samples in a solution of 1% Alcian Blue 8GX (10350, Electron Microscopy Sciences, Hatfield, PA, USA) in 3% acetic acid for 60 min. Tartrate-resistant acid phosphatase (TRAP) staining was performed using the TRAP/ALP Staining Kit (294-67001, Fujifilm Wako Pure Chemical Corporation, Osaka, Japan) according to the manufacturer’s instructions.

The stained sections were scanned and digitally converted into virtual slides using a Hamamatsu NDP slide scanner (NanoZoomer^®^ S20; Hamamatsu Photonics K.K., Hama matsu, Japan). The captured images were observed using image viewing software (2.29, NDP.view2, U12388-01, Hamamatsu Photonics K.K.).

## 3. Results

### 3.1. Stereoscopic Observations During the Regeneration Process

**Figure 2:**
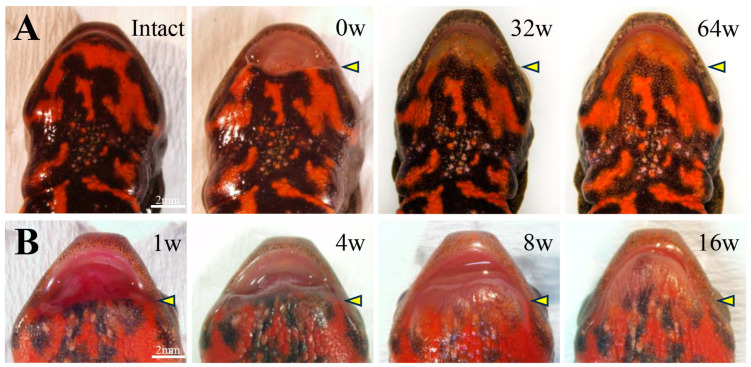
Stereoscopic images of the regeneration process after lower jaw amputation. An overview of the regeneration processes up to 64 weeks post-amputation (**A**) and images of early regeneration (**B**). The amputation site is indicated by an arrowhead. Scale bars: 2 mm.

Stereoscopic morphological changes to the amputated lower jaw were observed during the regeneration process up to 64 weeks (Figure 2A). By 32 weeks post-amputation, the lower jaw had regained its arched shape, nearly restoring its pre-amputation morphology. However, the black-and-orange mosaic pattern of the skin had not regained its original coloration, even at 64 weeks. Figure 2B shows the early stages of lower jaw regeneration. By 1 week post-amputation, the amputation margin was covered by transparent epithelium. By 4 weeks post-amputation, swelling developed throughout the entire wound site. By 8 weeks post-amputation, the regenerated area became clearly visible and expanded anteriorly. By 16 weeks post-amputation, the lower jaw contour had become arched.

### 3.2. Three-Dimensional Analysis of Lower Jaw Bone Regeneration Using Micro-CT

**Figure 3:**
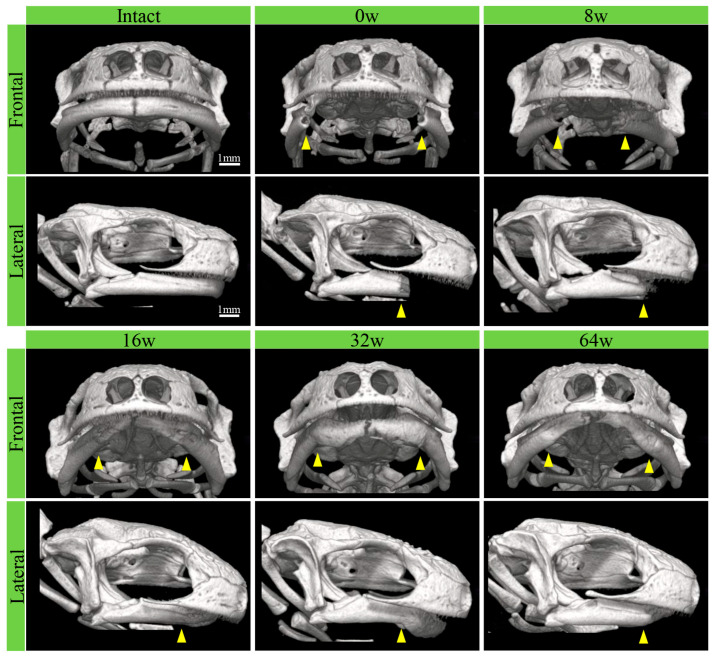
Micro-CT images of the craniofacial region (frontal and lateral views) from pre-amputation and immediately post-amputation through 64 weeks post-amputation. The amputation site is indicated by an arrowhead. Scale bars: 1 mm.

The morphological changes during regeneration after amputation, from pre-amputation through 64 weeks post-amputation, were analyzed using micro-CT (Figure 3). Micro-CT images of the entire craniofacial region clearly show the relative positions of the upper jaw bone and lower jaw bone (frontal and lateral views). The pre-amputation lower jaw bone is visible from the midline suture to the temporomandibular joint region. The outer edge of the lower jaw arch closely matches that of the upper jaw. By 8 weeks post-amputation, new bone formation was observed at the amputation margin and progressed toward the midline by 16 weeks. At this stage, the lower jaw arch was still smaller than the intact one. By 32 to 64 weeks post-amputation, the regenerated lower jaw arch extended to a position nearly equivalent to that of the intact arch. No significant changes were observed in the upper jaw bones.

**Figure 4:**
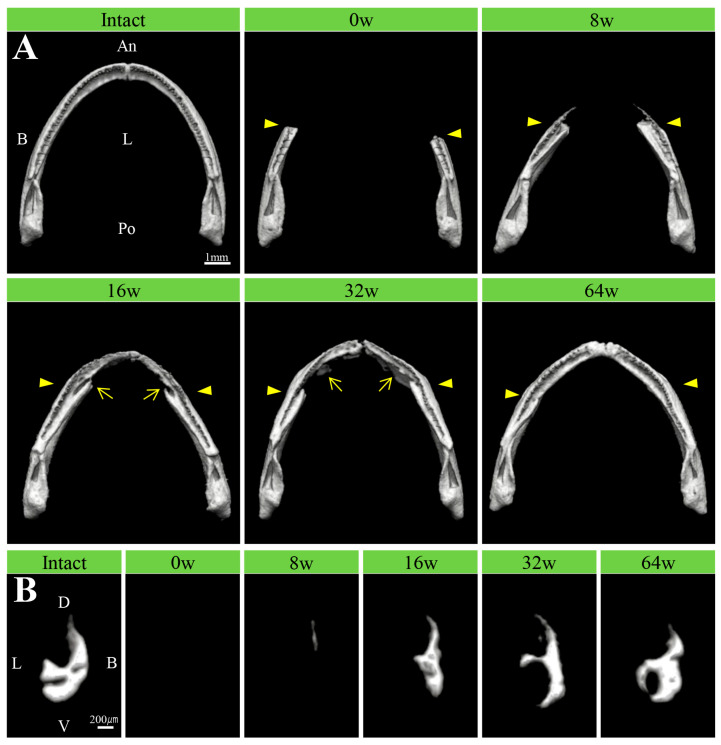
Micro-CT images of the regenerated lower jaw bones from pre-amputation and immediately post-amputation through 64 weeks post-amputation. (**A**) The overall dorsal-lateral view of the lower jaw bones is isolated via computer processing. The arrows indicate bone regeneration on the lingual aspect of the lower jaw arch. The amputation site is indicated by an arrowhead. An, anterior; B, buccal; L, lingual; Po, posterior. Scale bar: 1 mm. (**B**) Computer-trimmed transverse sectional view of the lower jaw bone at the midpoint between the amputation line and the midline. D, dorsal; V, ventral. Scale bar: 200 µm.

The regeneration process of the lower jaw bone is clearly shown by excluding the bones of the head and maxillary region (Figure 4A). New bone observed from 8 weeks extended anteriorly as thin bone from the buccal side of the bone amputation margin, while no new bone formation was observed on the lingual side of the lower jaw bone. This new bone corresponded to the region where teeth form. By week 16, the addition of bone to the lingual side of the lower jaw began, and the bone became slightly thicker. By week 32, the addition of bone to the lingual side was pronounced. This anterior extension increased the lower jaw bone thickness. The shape of the lower jaw arch also continued to extend toward the midline. By week 64, the lower jaw arch shape, the lower jaw bone, and the dentition were nearly restored to their pre-amputation state.

Transverse sectional views of the regenerating lower jaw bone showed new bone formation only on the buccal side of the lower jaw bone between 8 and 16 weeks after amputation. However, on the lingual side, bone addition began at 32 weeks and increased to a thickness nearly equivalent to that before amputation by 64 weeks (Figure 4B).

### 3.3. Morphometric Analysis of Regenerated Lower Jaw Bone

**Figure 5:**
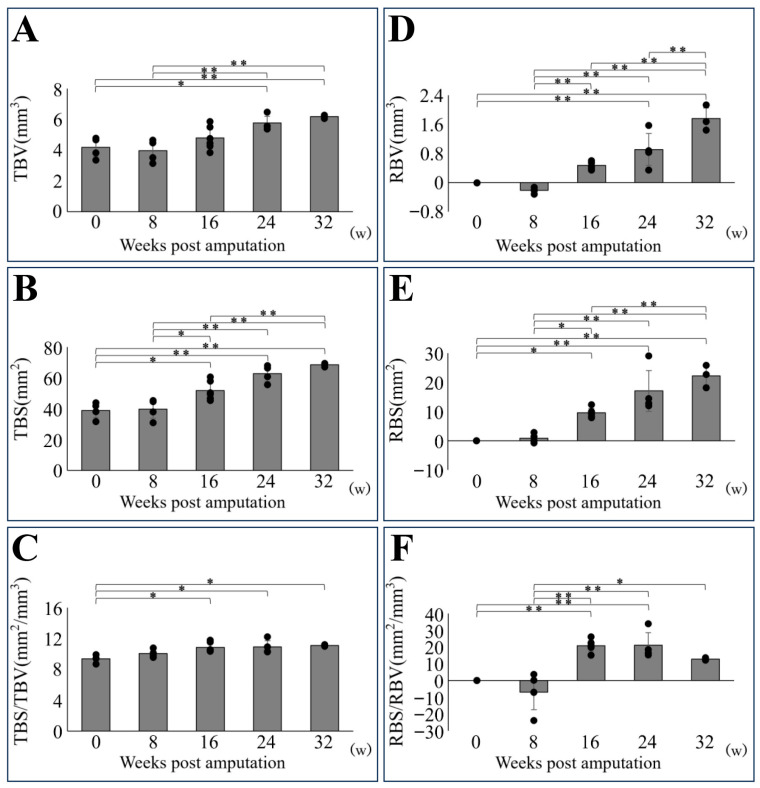
The morphometry of the regenerated lower jaw bones up to 32 weeks after amputation. The graphs on the left show results for the entire lower jaw, including existing and regenerated bones (**A**–**C**). The graphs on the right show the results for regenerated bones only (**D**–**F**). (**A**,**D**): bone volume; (**B**,**E**): bone surface area; (**C**,**F**): bone surface-to-volume ratio. Bone volume: TBV (total bone volume), RBV (regenerative bone volume); bone surface area: TBS (total bone surface area), RBS (regenerative bone surface area); bone surface-to-volume ratio: TBS/TBV (total bone surface-to-volume ratio), RBS/RBV (regenerative bone surface-to-volume ratio). * *p* < 0.05, ** *p* < 0.01.

To quantify both total bone parameters, including regenerated and existing bone and regenerated bone parameters alone, morphometric measurements of BV, BS, and the BS/BV were performed (Figure 5). Measurement of total bone parameters showed a decrease at 8 weeks post-amputation, followed by an increase in TBV over time (Figure 5A). TBV at 24 weeks and beyond differed significantly from TBV immediately post-amputation and at 8 weeks post-amputation. TBS increased over time and showed a significant difference from 16 weeks onward (Figure 5B). TBS/TBV gradually increased over time and also showed a significant difference from 16 weeks onward (Figure 5C).

Measurement of regenerative bone parameters alone revealed that RBV initially decreased, exhibiting a negative value at 8 weeks post-amputation. The negative values obtained are due to bone loss caused by bone resorption, as described below, rather than errors. However, values subsequently increased over time (Figure 5D). RBV showed a significant increase from immediately after amputation at 24 weeks and beyond, with significant differences observed between individual time points from 8 weeks onward. RBS exhibited an upward trend over time, demonstrating a significant difference compared with the immediate post-amputation period, beginning at 16 weeks (Figure 5E). RBS/RBV decreased initially and exhibited a negative value at 8 weeks post-amputation. However, it subsequently exhibited nearly identical peaks at 16 and 24 weeks, followed by a decrease at 32 weeks (Figure 5F). Significant differences were observed at 16 and 24 weeks compared with the immediate post-amputation period.

### 3.4. Bone Resorption at the Amputation Site Prior to Bone Regeneration

**Figure 6:**
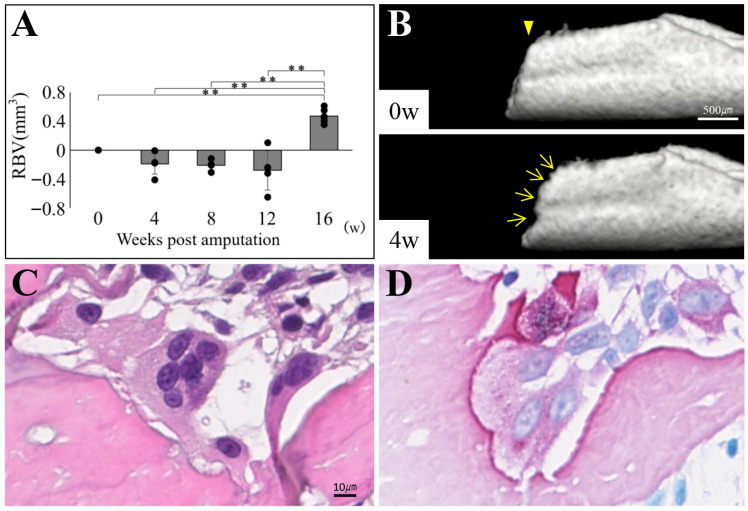
Verification of bone volume reduction prior to regeneration observed via morphometric analysis of regenerated lower jaw bones. Lower jaw bone volume decreased from week 4 to week 12 (**A**). Resorption pits were observed at the bone amputation margin on micro-CT images (**B**). The amputation site is indicated by an arrowhead. The arrows indicate the bone resorption pits. Scale bar: 500 µm. In hematoxylin and eosin (HE)-stained (**C**) and TRAP-stained (**D**) sections, TRAP-positive multinucleated osteoclasts were observed within the bone resorption pit. Scale bar: 10 µm. ** *p* < 0.01.

The reason for the decrease in BV immediately before regeneration, as shown in the bone morphometry results in Figure 5, was examined (Figure 6). Detailed bone morphometric analyses of the period up to 16 weeks post-amputation revealed that the mean RBV value was negative from weeks 4 to 12 (*p* < 0.01) (Figure 6A). Micro-CT images of the bone amputation margin in the same individual revealed that the amputation margin showed greater resorption at 4 weeks compared with immediately after amputation (Figure 6B). Histological sections of the amputation margin showed TRAP-positive multinucleated osteoclasts at 2 weeks (Figure 6C,D).

### 3.5. Regeneration of Bone and Cartilage in Lower Jaw After Amputation

**Figure 7:**
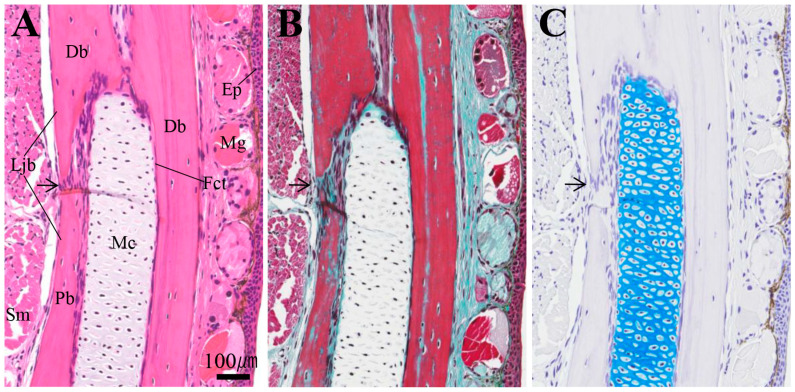
Histological structures surrounding the prospective lower jaw amputation site of an intact newt. From left to right: (**A**) HE, (**B**) MG, and (**C**) AB staining. The top of the tissue section is in the distal direction, the right is in the buccal direction, and the left is in the lingual direction (histological images in subsequent figures are oriented the same way). The arrows indicate the joint between the dentary and prearticular bones. Scale bar: 100 µm.

Figure 7 shows the tissue appearance near the prospective amputation area of the lower jaw in the intact newts. The amputation site in the anterior half of the lower jaw is located slightly anterior to the distal end of the prearticular bone (Supplementary Figure S1). On this histological section, Meckel’s cartilage, stained positive for AB, runs parallel between the buccal and lingual sides of the lower jaw bone via fibrous connective tissue. The lower jaw is a dense lamellar bone, and no structure corresponding to cancellous bone was observed.

**Figure 8:**
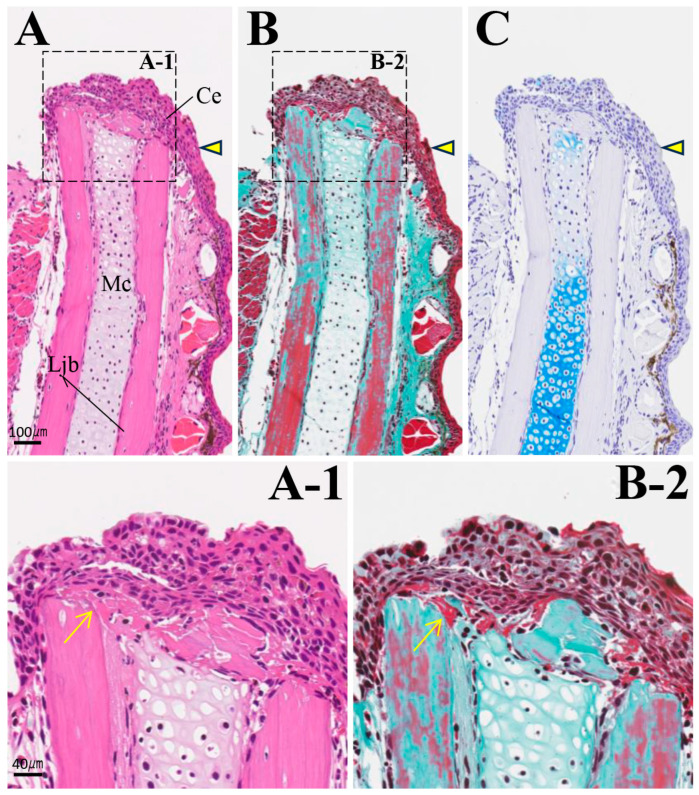
Histology of the amputation site at 2 weeks post-amputation. The amputation site is indicated by an arrowhead. Scale bar: 100 µm. (**A-1**) and (**B-2**) are enlarged images of the framed areas in (**A**) and (**B**), respectively. (**C**) AB staining. The arrows indicate fibrin deposited between the covering epithelium and the amputation margin. Scale bar: 40 µm.

**Figure 9:**
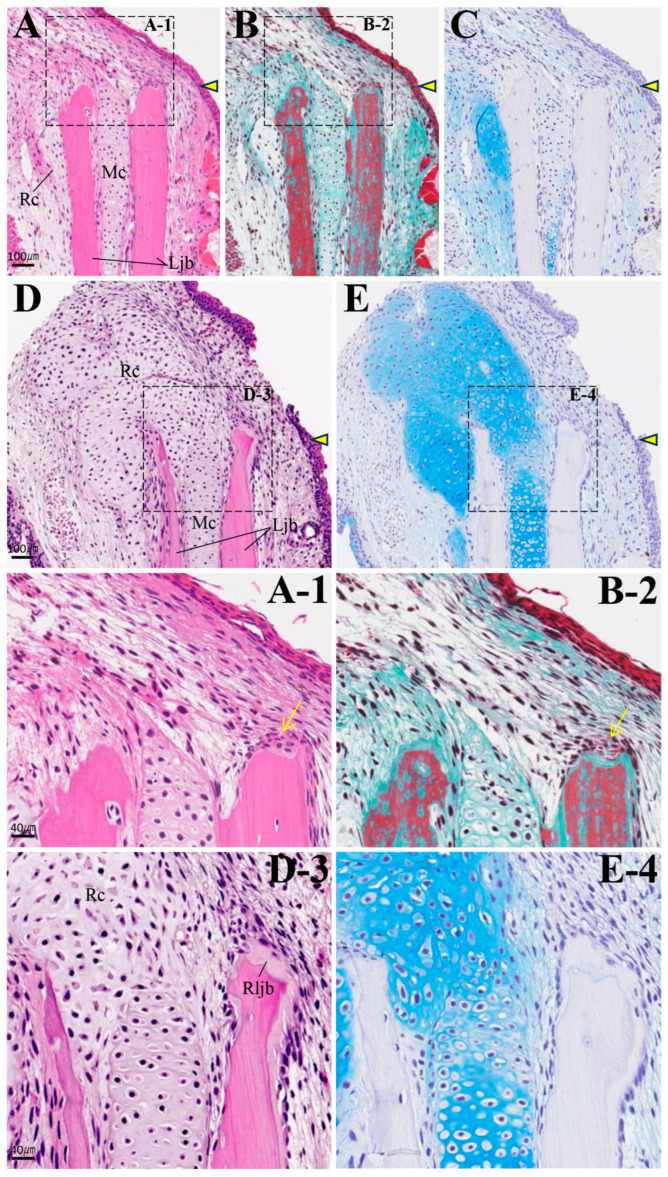
Histology of the amputation site at 4 weeks post-amputation. The individuals in (**D**) and (**E**) show more advanced regeneration than those in (**A**–**C**). The amputation site is indicated by an arrowhead. Scale bar: 100 µm. (**A-1**,**B-2**,**D-3**,**E-4**) are enlarged images of the square frames in (**A**), (**B**), (**D**), and (**E**), respectively. The arrows indicate the adhered osteoblasts. Scale bar: 40 µm.

By 2 weeks post-amputation, the amputation margin was covered by wound epithelium, which thickened and became stratified (Figure 8A–C). Fibrin, stained red via MG staining, was deposited between the epithelium and the amputation margin. In Meckel’s cartilage near the amputation margin, AB staining intensity decreased, and it exhibited a mild green color with MG staining. By 4 weeks, mesenchymal cells appeared between the covering epithelium and the amputation margin. These mesenchymal cells exhibited nuclear enlargement and varied in size and shape (Figure 9A,B). Interestingly, on the continuous section, it was confirmed that a small AB-positive cartilage formed at the most lingual position of the lower jaw bone, independent of the location of Meckel’s cartilage (Figure 9C). At the amputation site on the buccal side of the lower jaw bone, resorption pits were observed, and osteoblasts adhered to the surface of these resorption pits. However, no osteoblasts were detected on the lingual side of the lower jaw bone.

**Figure 10:**
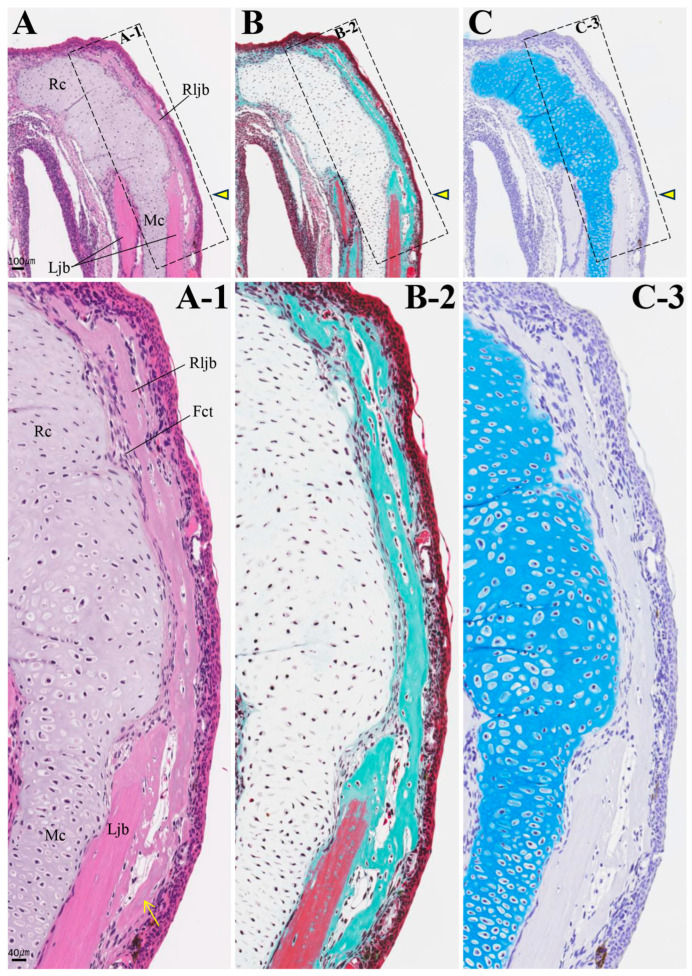
Histology of the amputation site at 8 weeks post-amputation. Scale bar: 100 µm. (**A-1**), (**B-2**), and (**C-3**) are enlarged images of the framed areas in (**A**), (**B**), and (**C**), respectively. The amputation site is indicated by an arrowhead. The arrow indicates newly formed bone added to the lateral aspect of the existing lower jaw bone. Scale bar: 40 µm.

**Figure 11:**
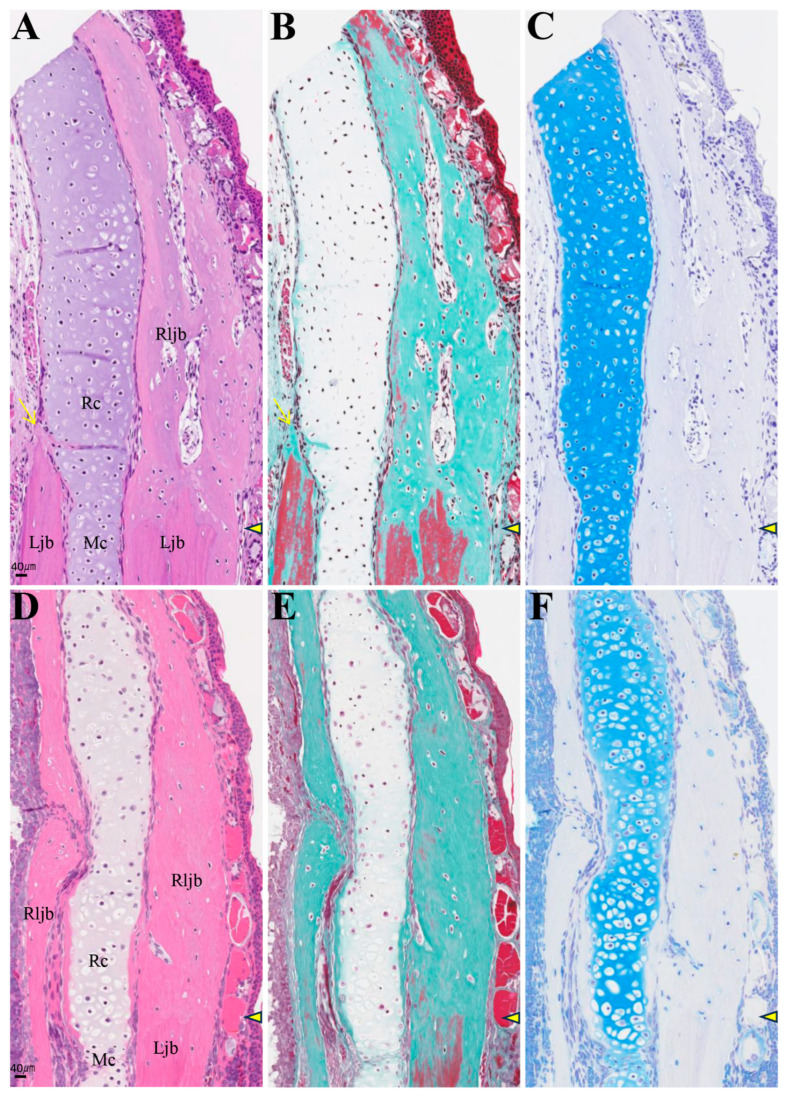
Histology of the amputation site at 16 (**A**–**C**) and 32 weeks (**D**–**F**) post-amputation. The amputation site is indicated by an arrowhead. The arrows indicate newly formed bone in the lingual side. Scale bar: 40 µm.

**Figure 12:**
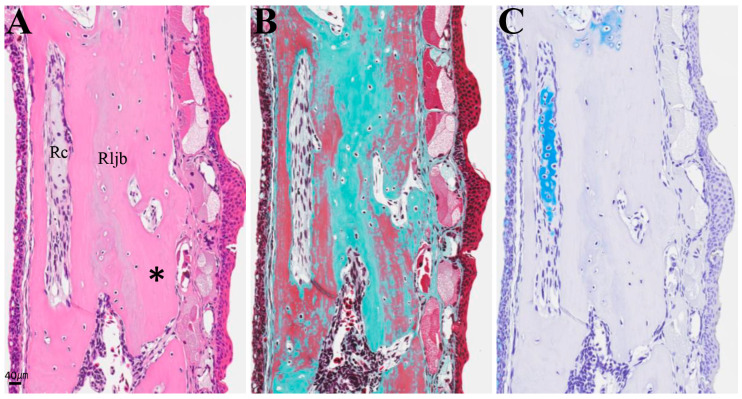
Histology near the area of the amputation at 64 weeks post-amputation. From left to right: (**A**) HE, (**B**) MG, and (**C**) AB staining. The bone structure of the lower jaw restored to its pre-amputation state. The asterisk indicates the lamellar structure of the bone. Scale bar: 40 µm.

In individuals with accelerated regeneration during the same period, cartilage proliferation was pronounced and merged with Meckel’s cartilage (Figure 9D,E). The AB stainability of the existing Meckel’s cartilage gradually improved. Osteoblasts adhered to the amputation surface on the buccal side of the lower jaw bone, and osteoid formation was also observed. By 8 weeks, not only was cartilage formation more pronounced, but newly formed bone originating from the buccal side of the bone amputation margin extended toward the anterior area (Figure 10). This new bone was fibrous, appearing green with MG staining, containing numerous lacunae filled with fibrous tissue, and exhibiting an irregular surface. The formation of this new bone was not accompanied by cartilage formation. At 16 weeks, regenerated new bone showed marked thickening on the buccal side, but bone formation slightly progressed on the lingual side (Figure 11). The regenerated cartilage became thinner and changed to a shape resembling the original Meckel’s cartilage. At 32 weeks, lower jaw bone formation became pronounced, even on the lingual side. By week 64, lower jaw bone formation progressed increasing in density and restoring the lower jaw bone to its pre-amputation thickness (Figure 12). The regenerated bone, which had previously been fibrous, developed a lamellar structure.

### 3.6. Tooth Regeneration After Amputation

**Figure 13:**
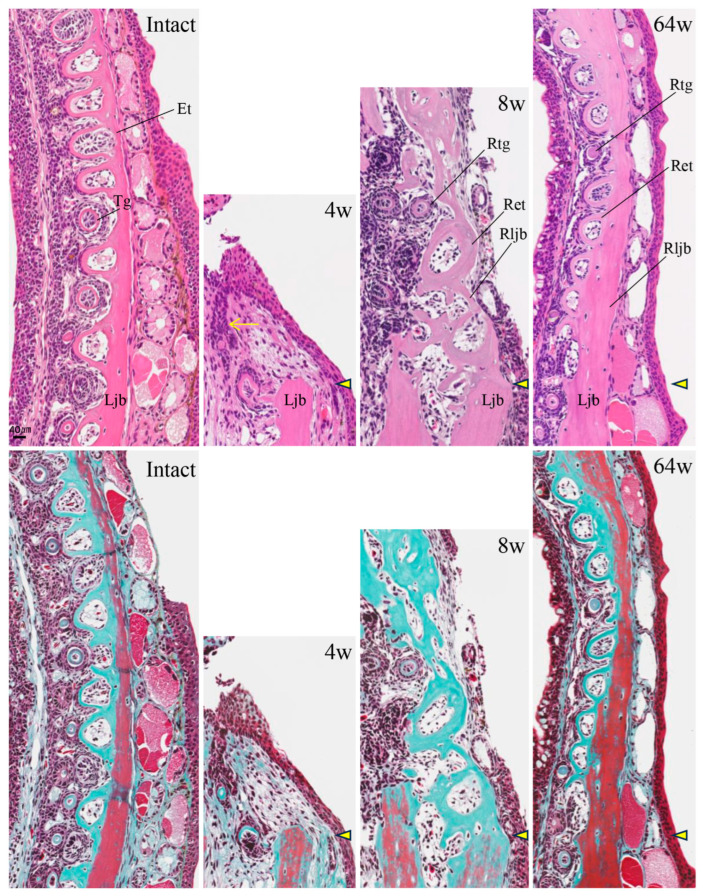
HE and MG staining images of the tooth regeneration process up to 64 weeks post-amputation and in intact animals. Tooth regeneration began early after amputation and continued the existing dentition. The amputation site is indicated by an arrowhead. The arrow indicates the dental lamina. Scale bar: 40 µm.

In intact newts, numerous teeth were neatly arranged along the lower jaw bone (Figure 13). Due to polyphyodonty, on horizontal sections, dental lamina and tooth germs formed at the tips of the dental lamina were arranged from the lingual to the buccal side, and erupted teeth were connected to the lower jaw bone. By 4 weeks, prior to lower jaw bone formation, the dental lamina invaginated from the covering epithelium, and the tooth germ at its tip was confirmed. By 8 weeks, tooth germs and erupting teeth were observed forming adjacent to irregularly regenerated lower jaw bone near the amputation site, continuing from the existing dentition. By 64 weeks, numerous tooth germs and erupted teeth were seen extending nearly to the midline, restoring a dentition similar to that prior to amputation.

## 4. Discussion

This study examined the regeneration of lower jaw tissues, particularly the hard tissues of bone and cartilage, following the amputation of the anterior half of the lower jaw, up to 64 weeks post-amputation. Stereoscopic observation suggested that the lower jaw arch restored its shape around 16 weeks post-amputation. However, analysis using micro-CT and histological sections revealed that lower jaw bone regeneration actually required 64 weeks. Previous reports on lower jaw amputation in amphibians have examined only up to 26 weeks post-amputation [4,5,11], and none analyzed the completion of hard tissue regeneration as in this study. In our study, by week 16, the thickness of the buccal region of regenerating lower jaw bone increased, and bone regeneration of the lingual region began. By week 64, the bone structure was nearly equivalent to that before amputation. Ghosh et al. [5] stated that bone regeneration does not occur in the lingual region, but this is likely because their observation period was limited to a maximum of 20 weeks. Our research revealed for the first time that even after an anterior half amputation of the lower jaw, the entire lower jaw bone regenerates from the buccal to lingual side.

In lower jaw bone regeneration, histological observations revealed that bone formation occurs directly by osteoblasts without undergoing cartilage formation. These findings suggest that bone regeneration occurs via membranous ossification. During development, the lower jaw bone is formed by membranous ossification, a process in which neural crest-derived osteoblasts undergo direct ossification [12], and lower jaw bone regeneration follows the same ossification pattern as the bone-formation process during development. In fact, our preliminary real-time polymerase chain reaction (PCR) analysis revealed that expression of an osteoblast differentiation (bone formation) marker *runx2* peaked during the new bone formation phase (4 weeks post-amputation) and remained elevated thereafter (Supplementary Figure S2 and Table S2). The pattern of membranous ossification in bone regeneration of the lower jaw is common not only in newts but also in axolotls [4,5,13,14]. Furthermore, this study indicated that newly regenerated bone is fibrous bone and requires approximately 64 weeks to develop a lamellar structure.

Osteomorphometric analysis revealed that the BV of the lower jaw decreased temporarily around 8 weeks post-amputation, then increased from 16 weeks onward. This temporary reduction in BV was found to result from TRAP-positive multinucleated osteoclasts adhering to the amputation margin and actively resorbing bone. It has been reported that osteoclast-mediated bone resorption occurs even during limb regeneration in axolotl [15], but bone resorption during jaw bone regeneration in amphibians has never been reported until now. Furthermore, BS/BV measurements showed a temporary decrease at 8 weeks post-amputation, followed by significant increases at 16 and 24 weeks, and subsequently exhibited a decreasing trend. This was thought to be due to the reduction in the concavity of the regenerated bone and the smoothing of the bone surface observed in micro-CT and histological analyses from 32 weeks onwards.

Based on the findings of this study and considering survival strategies, the newly formed cartilage is considered to dominate the jaw regeneration area as a tactic to acquire predatory capabilities early. This allows the jaw to become functional sooner, bypassing time-consuming processes such as bone matrix formation and calcification. This temporary expansion of cartilage is presumed to have not only maintained jaw strength but also contributed to defining the jaw’s contour. This is because the cartilage provides a route for regenerating the buccal lower jaw bone along the outer edge, leading to restoration of the jaw arch shape. Additionally, our preliminary real-time PCR analysis revealed that expression of *sox9*, a chondrocyte differentiation (cartilage formation) marker, peaked during this period of cartilage expansion (4 weeks post-amputation) (Supplementary Figure S2 and Table S2). Furthermore, the earliest regenerated buccal thin lower jaw bone aligned with the tooth-forming region, suggesting it provides an early site for regenerating teeth necessary for predation. In addition, expression of *amelogenin*, an ameloblast differentiation (tooth formation) marker, showed a pattern of gradual increase starting from 8 weeks post-amputation, consistent with the histological onset of erupting tooth formation (Supplementary Figure S2 and Table S2).

As time passed, the newly regenerated buccal lower jaw bone thickened and gained strength. Concurrently, the expanded cartilage gradually narrowed, transforming into a thickness approximating that of the original Meckel’s cartilage. As the cartilage thinned, regeneration of the lingual lower jaw bone commenced, restoring it to a bone structure similar to that of the lower jaw bone prior to amputation. The process of regenerating hard tissues—bone, cartilage, and dentition—in the lower jaw demonstrates a sophisticated and efficient regenerative strategy. It involves a precise coordination between cartilage and bone regeneration, enabling earlier feeding capabilities and enhancing survival prospects.

This study has several limitations. First, the analyses were exploratory in nature. Although a strict sample size calculation was not feasible for this type of study, the modest sample size warrants a cautious interpretation of the statistical results, and further validation in larger cohorts will be important. Second, because the genome of *Cynops pyrrhogaster* has not yet been fully characterized, PCR primer design relied on a closely related species. As a result, the precision of gene identification—and thus the extrapolation of molecular findings to humans—remains limited, though this limitation may improve as genomic resources for this species expand.

## 5. Conclusions

Our current research elucidated the regeneration process of the lower jaw in the Japanese fire-bellied newt. Specifically, the regeneration of the lower jaw bone, Meckel’s cartilage, and dentition was clarified. Furthermore, these regenerative processes were found to be closely linked to the newt’s survival strategy. It was demonstrated that newts can completely regenerate their jaws even after amputation. Overall, we believe that a deeper understanding of this mechanism may open the door to its application in humans, and we intend to advance this research to support the eventual realization of human jaw regeneration.

## Data Availability

All data used in this study are available from the corresponding authors upon reasonable request.

## Supplementary Materials

The following supporting information can be downloaded at: https://www.mdpi.com/article/10.3390/biomedicines14020434/s1, Figure S1: Illustration of the orientation of tissue sections; Figure S2: Results of real-time PCR analysis; Table S1: Summary of the number and classification of newt specimens analyzed in this study; Table S2: PCR primers used in this study; Supplementary Method for Real-time PCR Analysis.

## Abbreviations

The following abbreviations are used in this manuscript:

Ce	Covering epithelium
Db	Dentary bone
Ep	Epidermis
Et	Erupted tooth
Fct	Fibrous connective tissue
Tg	Tooth germ
Ljb	Lower jaw bone
Mc	Meckel’s cartilage
Mg	Mucosa gland
Pb	Prearticular bone
Rc	Regenerated cartilage
Ret	Regenerated erupted tooth
Rljb	Regenerated lower jaw bone
Rtg	Regenerated tooth germ
Sm	Skeletal muscle
