# Supplementary material for: Regeneration of Bone, Cartilage, and Tooth Following Lower Jaw Amputation in Newts
Source: Biomedicines. 2026 Feb 14;14(2):434. doi: 10.3390/biomedicines14020434 (PMC12938529; doi:10.3390/biomedicines14020434)
Supplement: Supplementary file 1 [file biomedicines-14-00434-s001.zip › biomedicines-4070892-supplementary.pdf]

Supplementary Information

for

Regeneration of Bone, Cartilage, and Tooth Following Lower Jaw Amputation in Newts

Kento Tsubosaki, Taisuke Hani, Kazuya Fujita, Kaori Sato, Tomoo Kudo, Yuuichi Soeno, Tatsuyuki Ishii, Kazuo Kishi, Chikafumi Chiba, and Yuji Taya

CC: chichiba@biol.tsukuba.ac.jp

YT: taya-yu@tky.ndu.ac.jp

Supplementary Table S1. Summary of the number and classification of newt specimens analyzed in this study

Time points	Total numbers of newt	Stereo microscopy	Micro-CT (osteomorphometry)	Histological observation	qPCR
Control (intact)	**7**	7	7	3	1
0 w	**3**	3	3	2	0
1 w	**8**	8	8	3	0
2 w	**8**	8	8	3	0
4 w	**9**	9	9 (4)	3 *	1
8 w	**9**	9	9 (4)	3 *	1
12 w	**6**	6	6 (4)	0	1
16 w	**9**	9	9 (6)	3 *	1
24 w	**6**	6	6 (4)	1	0
32 w	**6**	6	6 (3)	3 *	0
64 w	**3**	3	3	3	0

The initial study design specified observational and analytical time points at 4, 8, 16, and 32 weeks following lower jaw bone amputation. As the analyses progressed—particularly those concerning the dynamics of bone formation—these time points were refined to optimize data acquisition. Three individuals that died during the postoperative observation period were excluded from further analyses. Therefore, the total sample size is 77 individuals.

*For histological observation, at least one specimen was derived from an individual that had also undergone micro-CT–based morphometric evaluation.

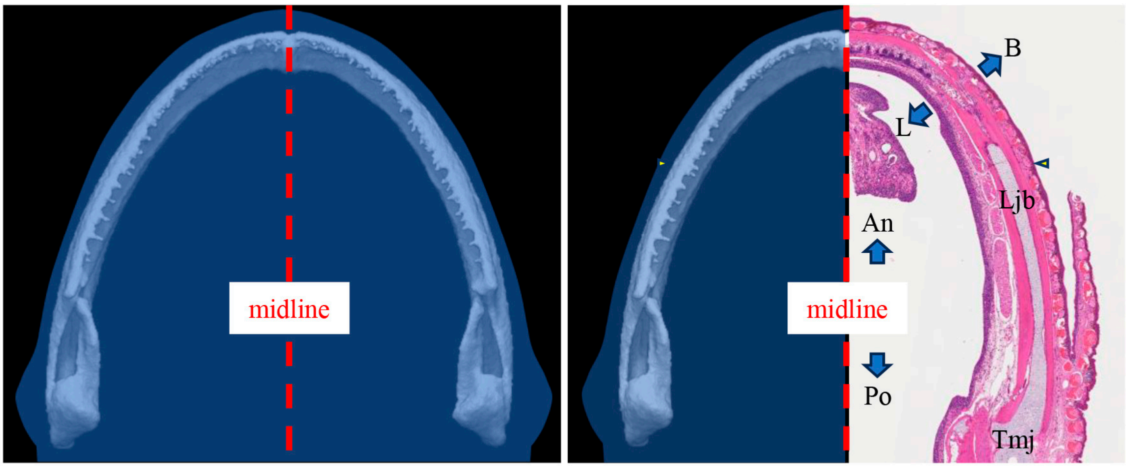

Supplementary Figure S1. This illustrates the orientation of tissue sections. The left diagram is a schematic overlaying soft tissue contours onto a micro-CT image of the intact lower jaw in dorsal-lateral view. The right diagram shows a horizontal section cut through the midline and indicates its orientation. An: anterior, Po: posterior, B: buccal, L: lingual, Ljb: lower jaw bone, Tmj: temporal mandibular joint. The prospective amputation site is indicated by an arrowhead.

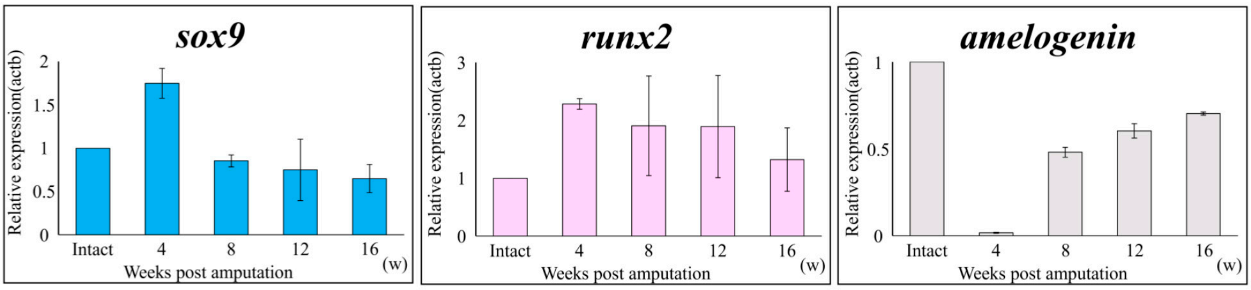

Supplementary Figure S2. Real-time PCR analysis results for gene expression related to bone, cartilage, and tooth formation markers in lower jaw regenerative tissue are presented. *sox9* was used as a marker for cartilage differentiation, *runx2* for osteoblast differentiation, and *amelogenin* for tooth formation (enamel protein production). The cut fragment from the amputation was used as a control. *sox9* and *runx2* showed peak expression at 4 weeks. *amelogenin* exhibited a trend of increasing expression chronologically. These expression patterns were consistent with histological findings on tissue sections.

Supplementary Method for real-time PCR analysis

In the amphibian newt *Cynops pyrrhogaster*, neither its genome nor gene sequences is known. Primer sets were designed using genetic information in the closely related species *Pleurodeles waltl*. Target gene sequences were first searched at NCBI for information on *Pleurodeles waltl*. Primer sets were designed using primer-BLAST based on the target gene sequences. The high specificity of each sequence in these primer sets was confirmed through BLAST. Using the *Cynops pyrrhogaster* NGS database, sequence regions that are similar to these primer sets were identified. The primer set for *Cynops pyrrhogaster* was modified from *Pleurodeles waltl* the sequences and used for Real-time PCR. Then the regenerated tissues after mandibular amputation in *Cynops pyrrhogaster* were collected. After total RNA extraction, primer suitability was verified through real-time PCR condition optimization and melting curve analysis. Subsequently, expression levels of target genes were quantified by real-time PCR. The PCR products were verified by electrophoresis to confirm band size. PCR products extracted from the target bands were inserted into a plasmid and sequenced. Analysis of the sequence confirmed it matched the target Japanese fire-bellied newt DNA sequence. Target genes were normalized against an internal control gene (*beta-actin*) and the expression level was calculated using the delta-delta CT method. For real-time RT-PCR, biological replicates n=1 (technical replicates n=3). These results are preliminary, but as this paper is exploratory research, we plan to deepen our analysis to elucidate the underlying mechanisms in future studies. This study was approved by the Committee for Genetic Recombination Experiments of the Nippon Dental University School of Life and Dentistry at Tokyo (approval numbers 2403-041).

Supplementary Table S2. PCR primers used in this study

Gene name	Reference sequence (*Pleurodeles Waltl*)	Primer Sequence (5'-3')
sox9	GenBank: EU872027.1	F- GCC TTA GAC TGC TCC CAA GA<br>R- CCC AAG CTG TAG TGC AGA GG
runx2	XM_069236101.1	F- CCA GGC CTA CCC AGC CTA CT<br>R-TCT GAA GCA CCT GAG AGG CG
amelogenin	GenBank: JX508595.1	F- GGC TAT GAA CCA ATG GGT GG<br>R- GTG TGG AAA ATG CTG CTG GG
beta-actin	GenBank: JG015872.1	F- CTT GAC TTG GCT GGT CGT GA<br>R- GGC TGT AGT TGT GAA GCT GT
